# Total Talus Allograft Transplantation With Subtalar Arthrodesis for Missing Talus: A Report of a Rare Case

**DOI:** 10.7759/cureus.67664

**Published:** 2024-08-24

**Authors:** Chieh Loh, Chin Horng Su, Kai-Chiang Yang, Chen-Chie Wang

**Affiliations:** 1 Department of Orthopedic Surgery, Taipei Tzu Chi Hospital, Buddhist Tzu Chi Medical Foundation, New Taipei City, TWN; 2 Department of Orthopedics, Asia University Hospital, Taichung City, TWN; 3 School of Dental Technology, College of Oral Medicine, Taipei Medical University, Taipei City, TWN; 4 Department of Orthopedics, School of Medicine, Tzu Chi University, Hualien City, TWN

**Keywords:** avascular necrosis of the talus, talu, subtalar arthrodesis, open dislocation, talus avascular necrosis, talus extrusion, osteochondral allograft transplantation

## Abstract

Post-traumatic missing talus is a rare and severe injury that often results in poor functional outcomes, with no consensus on the optimal treatment approach as strategies vary based on injury severity. We present the case of a 44-year-old male who sustained a missing talus following a high-energy motorcycle accident. After initial wound management and application of an external fixator, the patient underwent size-matched, fresh-frozen talus allograft transplantation combined with subtalar fusion. Postoperative radiography and CT confirmed successful transplantation with solid subtalar fusion, although progressive osteonecrosis was noted in the medial shoulder region of the talus. At the two-year follow-up, the patient exhibited limited ankle and hindfoot motion but was able to bear weight and walk without assistance, reporting no pain in his feet and achieving a final American Orthopaedic Foot & Ankle Society hindfoot score of 72. This case underscores the potential of total talar allograft transplantation with subtalar arthrodesis in treating severe talar bone loss or missing talus, although long-term follow-up is necessary to assess the clinical implications of medial talar collapse and the possible need for revision surgery.

## Introduction

Post-traumatic missing talus, or total talar extrusion, is a rare injury typically resulting from high-energy trauma. This injury often occurs alongside peri-ankle fractures, open wounds, and other musculoskeletal injuries, leading to poor functional outcomes such as avascular necrosis of the talus and infection [1]. Due to its rarity, there is no established consensus on the optimal treatment for total talar extrusion. Treatment approaches vary depending on the severity of the injury and may include external fixation with staged talus reimplantation, direct talus reimplantation, tibiocalcaneal or tibiotalocalcaneal fusion, and total talar prosthesis replacement [2-5]. Fresh-frozen allograft transplantation has shown promising results in treating large osteochondral defects of the talus [6,7], but the use of whole talus allograft transplantation for talar extrusion is less commonly explored. We present a case of a missing talus treated with size-matched talus allograft transplantation combined with subtalar fusion.

## Case presentation

Our patient was a 44-year-old man who sustained a motorcycle accident resulting in a right medial ankle laceration and an inability to bear weight on his injured foot. He visited the emergency department of a local hospital, where medical staff noted the “missing talus.” The talus was eventually recovered 30 minutes later by a friend at the scene but was severely contaminated and discarded.

**Figure 1:**
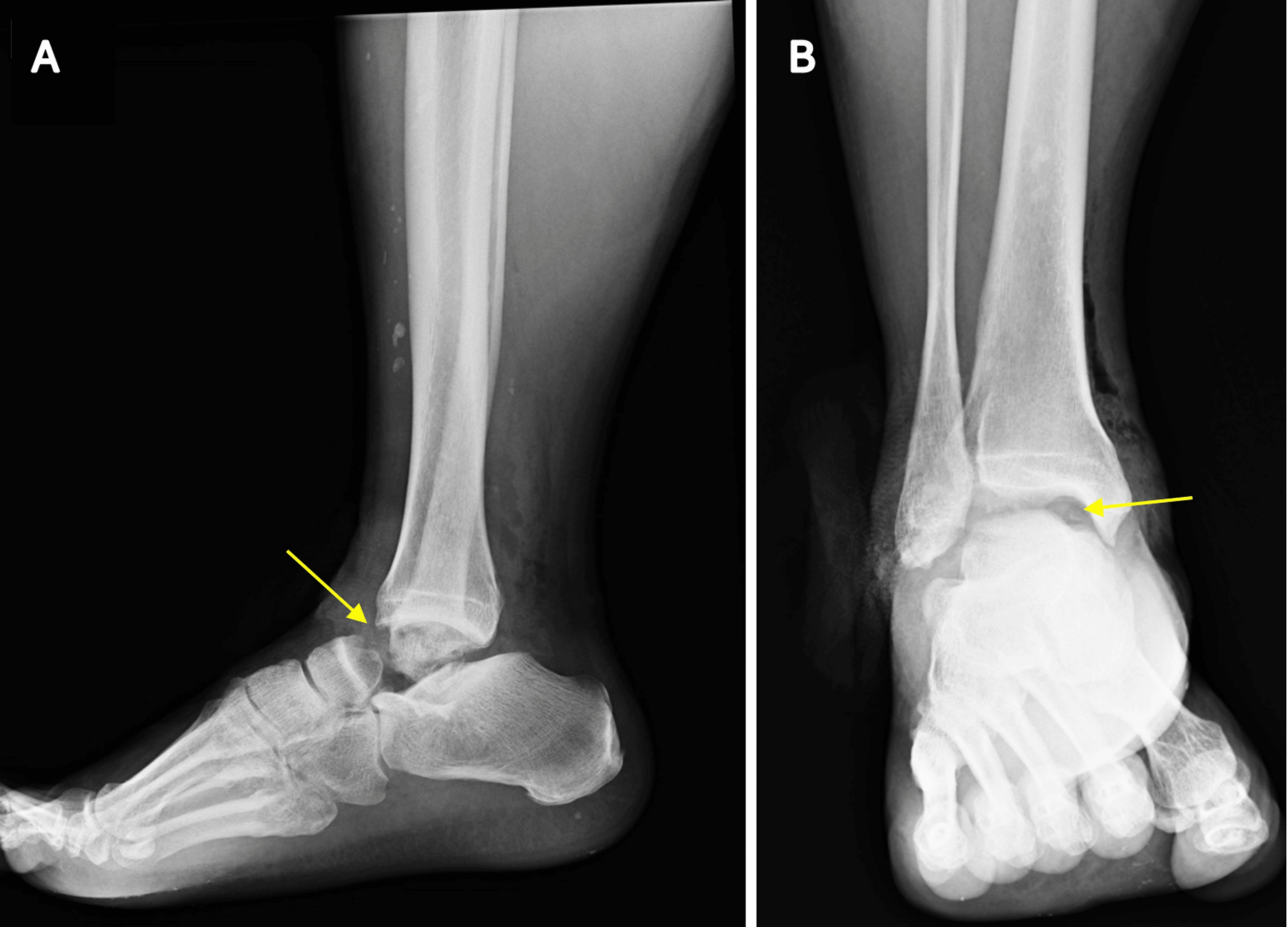
“Missing talus” (yellow arrow) with no associated peri-ankle fractures on (A) lateral and (B) AP views

**Figure 2:**
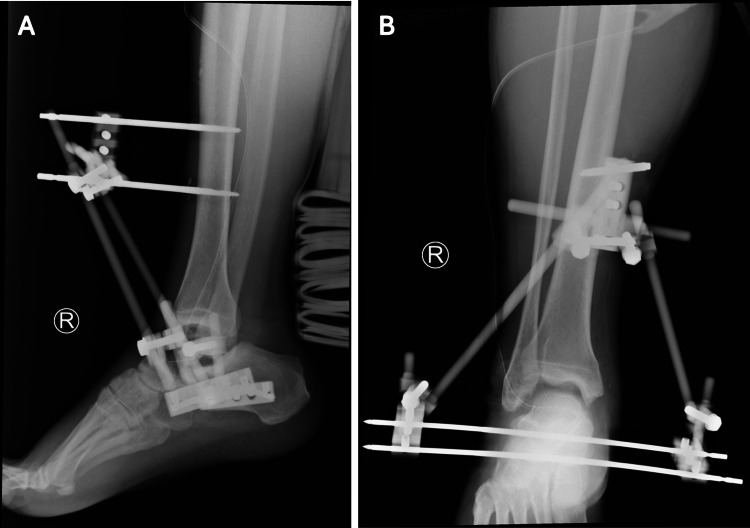
(A, B) External fixation following wound debridement and closure

The local hospital’s medical records documented a physical examination revealing a 9-cm medial ankle laceration without damage to major neurovascular structures. Radiographs of the right foot and ankle confirmed the missing talus and showed no associated fractures around the ankle and foot (Figure 1). The patient was referred to a tertiary medical center due to the complexity of the injury and did not undergo any initial surgical procedures. Cefazolin and gentamicin were administered for antibiotic prophylaxis, followed by immediate wound closure after debridement and irrigation within six hours of the injury. The antibiotic regimen continued for two weeks post-debridement. External fixation was applied in a triangular configuration to maintain the alignment and length of the ankle joint (Figure 2) and was performed concurrently with the debridement. No further wound complications were observed after the initial management.

With the soft tissue condition substantially improved, the patient was offered a custom-made ceramic total talus replacement based on a CT of the contralateral talus. However, the Taiwan Food and Drug Administration (TFDA) declined the application for compassionate use of the ceramic prosthesis. Due to the unavailability of TFDA approval for the total talar prosthesis and the patient’s reluctance to undergo tibiotalocalcaneal arthrodesis, he was referred to our hospital six months after the injury for total talar allograft transplantation, augmented with subtalar fusion.

Surgical technique

**Figure 3:**
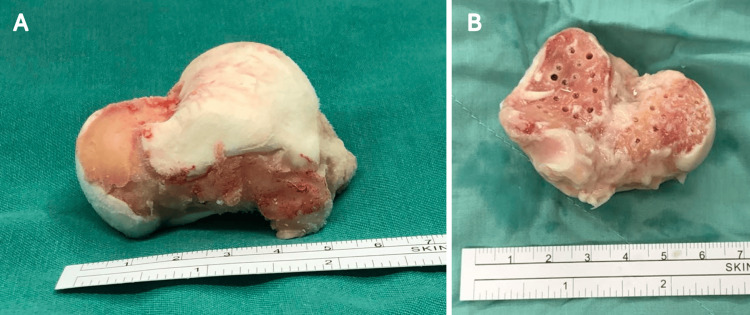
(A) Size-matched fresh-frozen talus allograft prepared for subtalar arthrodesis. (B) Multiple drillings were performed on the talus to enhance circulation through the anterior, middle, and posterior facets of the recipient calcaneus

**Figure 4:**
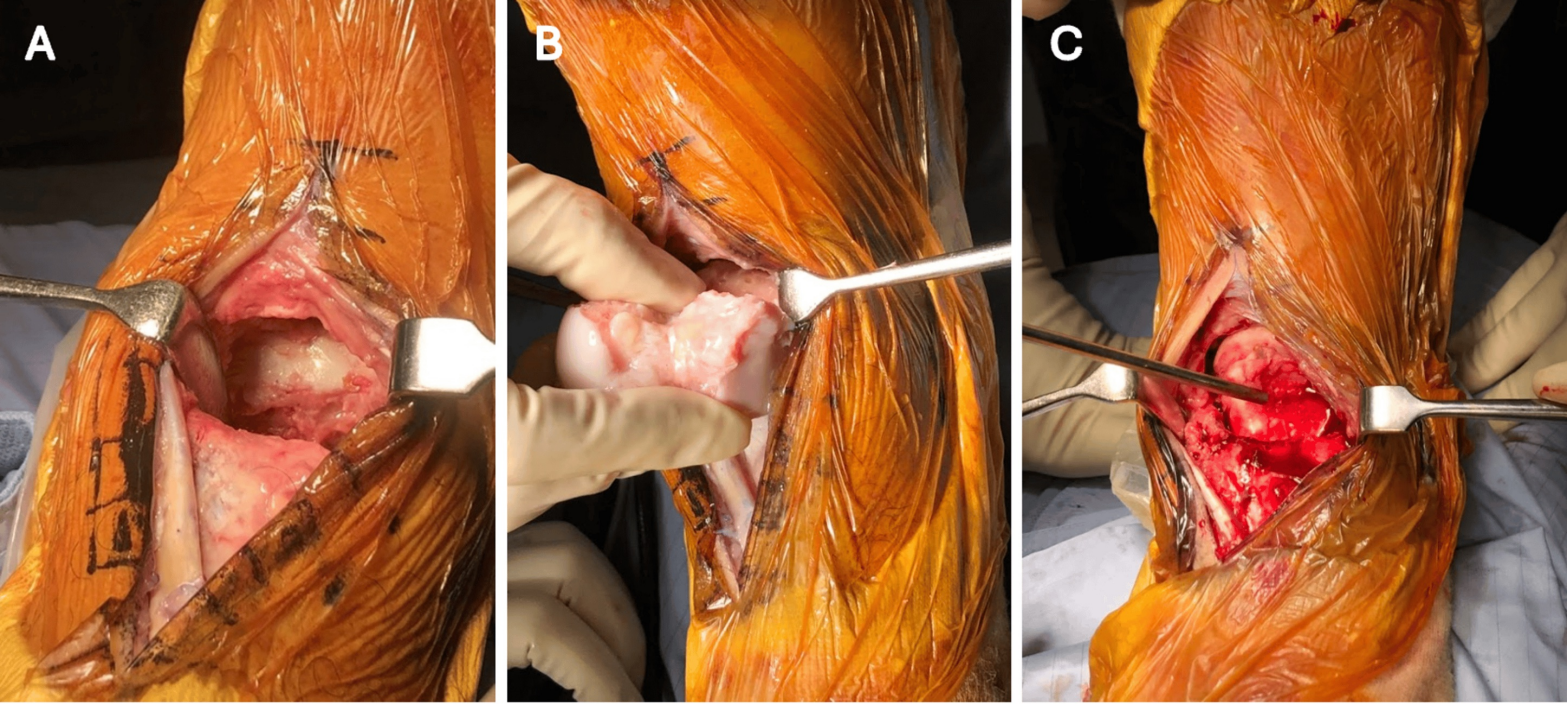
(A) Anterior approach to the ankle, followed by the (B, C) reduction of the prepared talus into position

**Figure 5:**
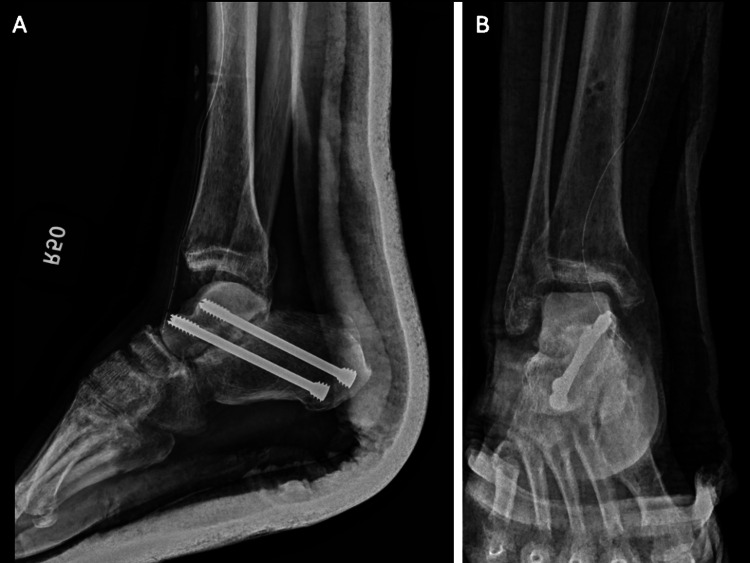
(A, B) Total talus transplantation augmented with subtalar arthrodesis

A size-matched fresh-frozen total talus allograft transplantation with subtalar arthrodesis was arranged six months after the initial trauma. Size-matching of the talus was performed by comparing the radiographs of the fresh-frozen allograft with the recipient’s preoperative contralateral ankle radiographs and CT scans. The external fixator was initially removed, and an anterior approach was used to expose the ankle joint. Fibrotic tissue within the bony vacancy was meticulously debrided to reveal the interior architecture between the tibial plafond and the upper surface of the calcaneus. Cartilage over the subtalar joint at both the recipient and donor sites was thoroughly removed, and the fusion bed was carefully drilled and irrigated with saline (Figure 3). The talus was then reduced into the ankle joint through longitudinal traction and temporarily fixed with multiple K-wires (Figure 4). Under fluoroscopic guidance, subtalar fusion was performed using two 6.5-mm headless compression screws in a retrograde fashion (Figure 5).

**Figure 6:**
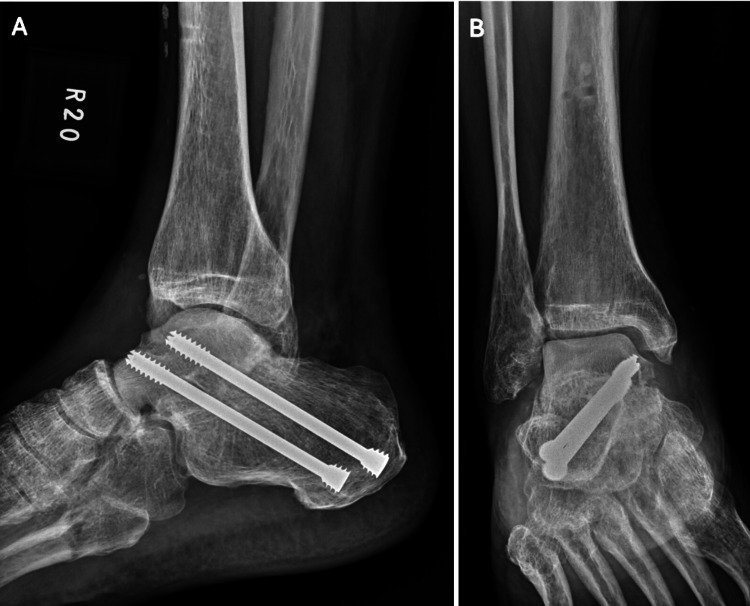
Solid subtalar fusion evident on both (A) lateral and (B) AP views, with no signs of talar avascular necrosis observed six months postoperation

**Figure 7:**
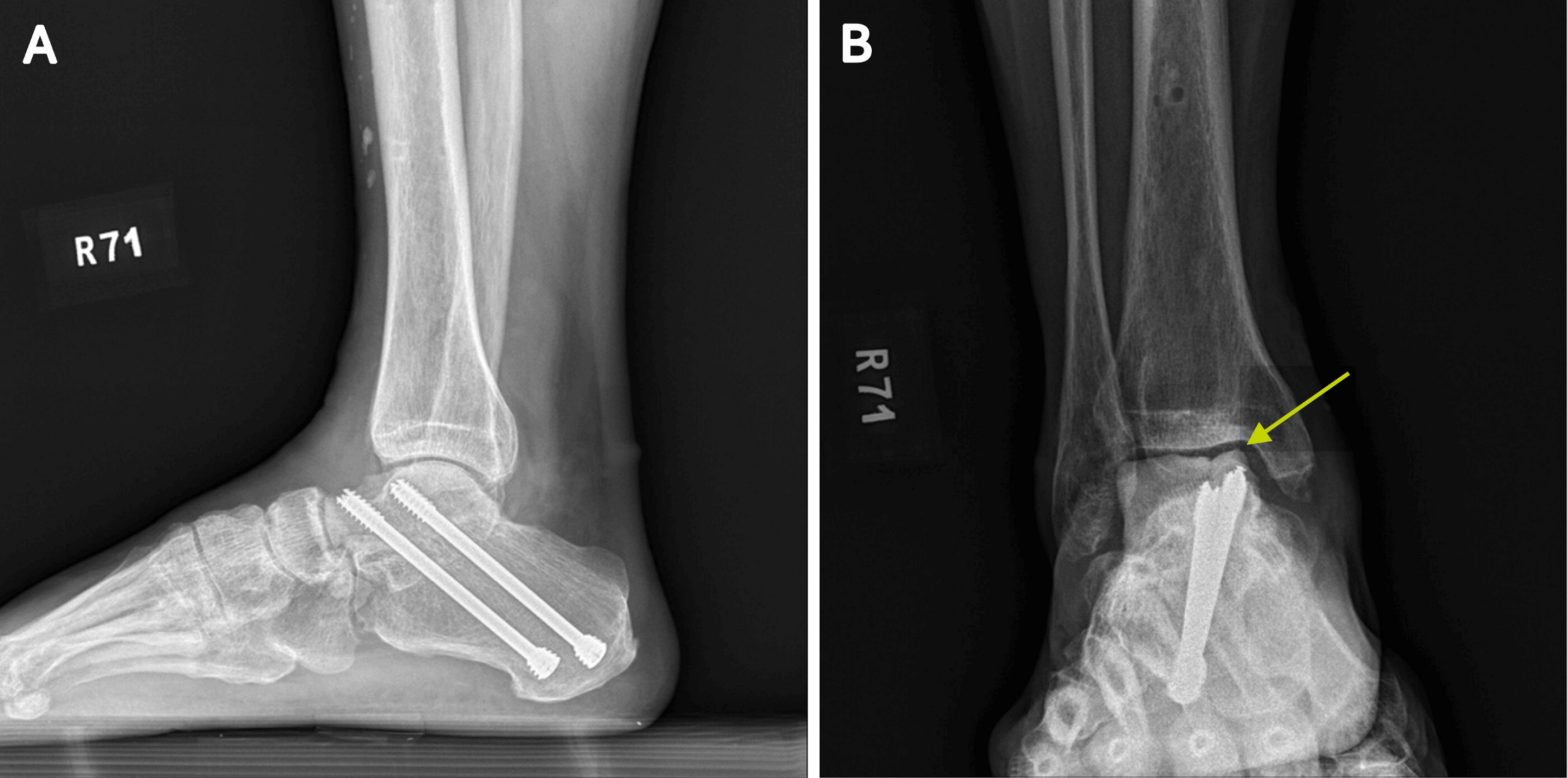
(A, B) Postoperative one-year radiographs revealed medial collapse (yellow arrow) of the transplanted talus allograft

**Figure 8:**
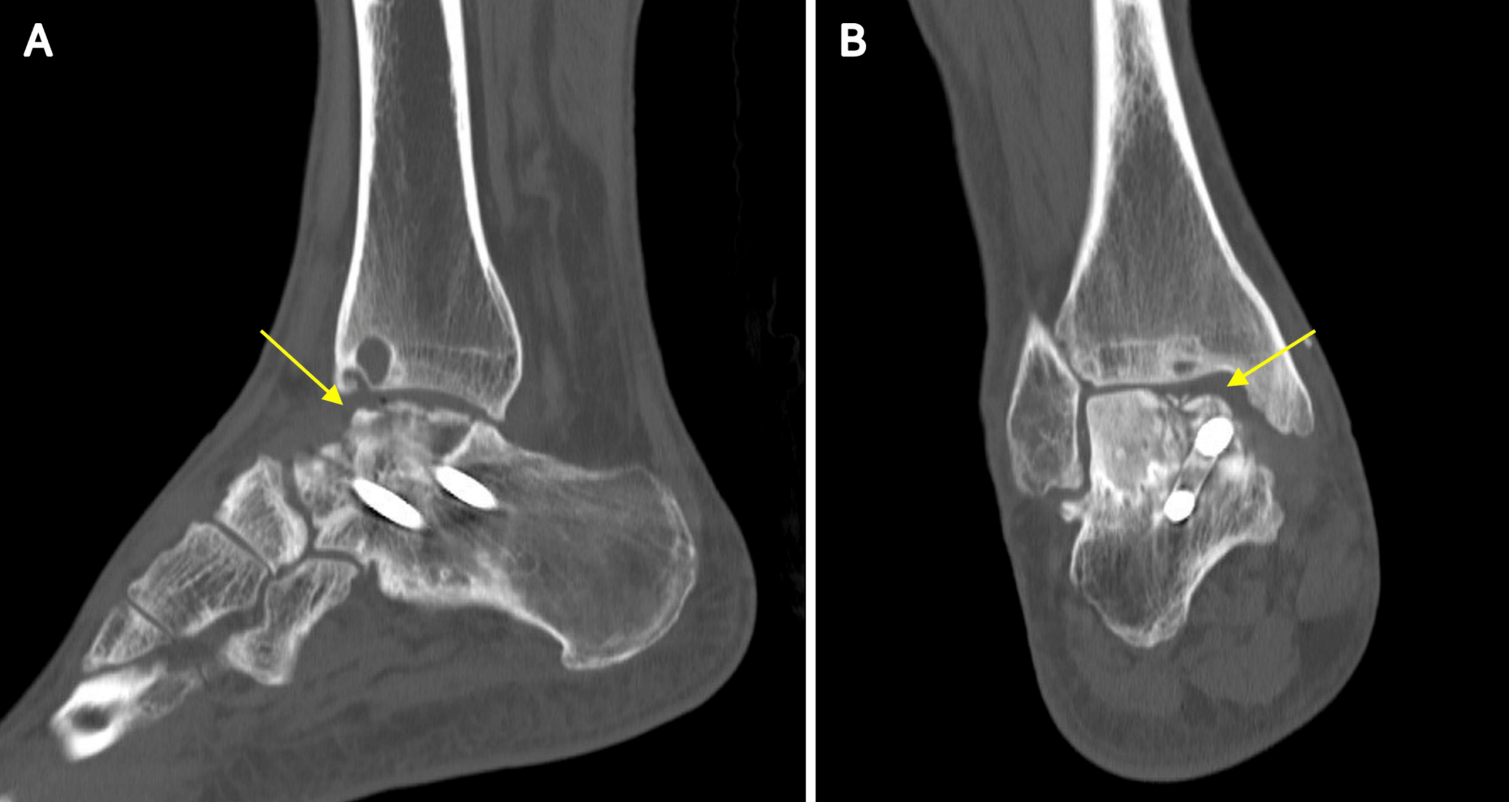
Postoperative three-year CT showed focal collapse (yellow arrow) on the medial side of the talus allograft, as seen in (A) lateral and (B) coronal views

No major complications were observed following the transplantation surgery. Postoperative radiographs of the right ankle showed successful transplantation of the talus with solid subtalar fusion at six months, without signs of loose hardware or infection (Figure 6). At the one-year follow-up, plain radiographs indicated progressive osteonecrosis of the talus in the medial shoulder region, but no further collapse was detected on the three-year postoperative CT scan (Figures 7, 8). Clinical examination revealed right ankle dorsiflexion and plantarflexion of 10 and 20 degrees, respectively, with severe hindfoot eversion and inversion, likely due to subtalar fusion and the prolonged use of an external fixator. Despite these limitations, the patient could bear weight and walk without crutches starting four months postoperation. Additionally, the patient experienced no pain during daily activities and regained pre-injury functional capacity of the foot by six months postoperation. The final American Orthopaedic Foot & Ankle Society hindfoot score was 72 at the two-year follow-up, and the patient expressed satisfaction with the functional outcomes.

## Discussion

Missing talus, or total talus extrusion, is a rare injury often resulting in significant functional disability. Traditionally, such injuries have been managed with tibiocalcaneal or tibiotalocalcaneal arthrodesis to minimize the risk of deep surgical site infections. However, recent studies have supported immediate talus implantation following wound debridement, showing favorable functional outcomes and acceptable complication rates [1]. A common complication is avascular necrosis, which results from inadequate vascular supply to the talus, necessitating revision surgeries and leading to functional deficits. While total talar replacement is a promising alternative, concerns about implant accessibility, the need for secondary surgery, and adjacent osteoarthritis remain [8].

Bulk allografts have been frequently utilized for treating osteochondral defects of the talus, with fresh-frozen allograft transplantation showing promising results for osteochondral lesions [7,9]. Despite this, whole talus transplantation is less commonly discussed in the literature. In our case, subtalar arthrodesis was employed to provide intraosseous blood supply to the avascular talus allograft, enhancing its survival [10]. Additionally, subtalar fusion stabilized the ankle joint, reducing shear forces on the talus allograft. However, focal avascular necrosis occurred, likely due to the patient’s smoking status and potential damage from the hardware, such as headless compression screws, affecting the medial side of the talus.

Fresh-frozen allograft transplantation reduces the risk of infection and immune reactions compared to fresh allografts. Although fresh allografts have higher chondrocyte viability, talus transplantation in our case yielded outcomes similar to those reported [11]. For cases of focal avascular necrosis of the transplanted talus, total ankle arthroplasty can serve as an alternative revision treatment, offering better functional outcomes than revision via ankle arthrodesis, especially for patients who have undergone subtalar fusion [12].

Postoperative ankle stiffness in our case may have been exacerbated by prolonged external fixation after the initial injury and the sacrifice of subtalar motion to improve talus viability. These factors contributed to less optimal outcomes following talar implantation. The lack of reconstruction of soft tissue attachments, such as lateral and medial ligaments, did not cause instability but may have contributed to the medial collapse of the talus allograft due to insufficient blood supply and circulation.

## Conclusions

Total talar allograft transplantation with subtalar arthrodesis yields favorable outcomes in patients with severe bone loss or a missing talus. This case report underscores the potential benefits of combining total talus transplantation with subtalar arthrodesis to enhance local circulation to the bone allograft. The patient experienced a satisfactory clinical outcome despite postoperative medial talus collapse. Long-term follow-up is essential to assess the clinical implications of medial talar collapse and to determine whether revision surgery may be necessary.
